# Prevalence and Factors Associated with Stunting among Public Primary School Pupils in Kasulu District, Western Tanzania.

**DOI:** 10.24248/eahrj.v4i2.641

**Published:** 2020-11-26

**Authors:** Jairos N. Hiliza, Leyna Germana, Amalberga Kasangala, Flora Joram

**Affiliations:** a Department of Epidemiology and Biostatistics Muhimbili University of Health and Allied Sciences; b Tanzania Field Epidemiology and Laboratory Training Programme; c Ministry of Health, Community Development, Gender, Elderly and Children; d Department of Community Development Studies Muhimbili University of Health and Allied Sciences

## Abstract

**Background:**

Underfeeding of a child in the first 2 years of life results in irreversible growth damage. Globally, stunting has declined from 39.7% in 1990 to 26.7% in 2010 while in Africa has remained at 40% since 1990. However, stunting is little known in primary pupils.

This study estimated the prevalence of stunting and contributing factors among public primary school pupils in Kasulu District.

**Method:**

Cross-sectional study was conducted among public primary pupils. Systematic random sampling was used to select study participants and then stratified to 5-7 and 8-12 years. Socio-economic factors, dietary practices, water, sanitation, and hygiene behaviours; school performance/attendance data were collected using a pretested questionnaire. Measurements were standardised to the World Health Organization HAZ-Scores for both girls and boys. Descriptive statistics, bivariate, and multivariable logistic regression were used to generate results.

**Results:**

A total of 400 pupils (100%RR) were recruited into the study, mean age of 7.51 (STD= 1.54) years and a half (50.3%) were boys. The prevalence of stunting was 127 (31.8%) (95% CI: 27.2%–36.6%), with no sex difference (63 (31.7%) – girls vs. 64 (31.8%) – boys; p = 0.969). Household wealth influenced stunting; lowest quintile (AOR= 28; 95% CI: 3.64 – 214.6; p<0.001) 2nd quintile (AOR = 17; 95%CI: 2.20 – 138.5; p<0.01), the 3rd quintile (AOR = 8.0; 95%CI: 0.99 – 64.67; p = 0.051) and 4th quintile (AOR = 4.2; 95%CI: 0.49 – 36.75; p = 0.191) when compared to 5th (highest) wealthquintile. Food insecurity (AOR = 10.6; 95%CI: 4.60 – 24.60; p< 0.001), less protein in meal were the risk for stunting (AOR = 14.6; 95%CI: 4.07 – 52.42; p<0.001). Inappropriate hand wash after toilets both at school, (AOR=3.5; 95%CI:1.62–7.58; p=0.001), and home (AOR = 13.0; 95%CI: 2.73 – 61.76; p = 0.001) were the risk for stunting. Stunted pupils had irregular school attendance (AOR = 9.4;95%CI: 4.42 – 19.93; p<0.001) and poor performance (AOR = 23.6; 95%CI: 10.24 –54.19; p<0.001). Food insecurity influenced poor performance (AOR = 3.9; 95%CI:1.67–8.92; p<0.01) and irregular school attendance (AOR=5.4, p=0.000).

**Conclusion:**

Stunting among public primary school pupils is very high despite the prevention effort. Low wealth, food insecurity, poor hand hygiene, and lack of protein in a meal significantly influence stunting. Also, it affects the pupils’ academic performance and attendance, availability of food in both quantity and quality, community nutrition

## INTRODUCTION

Adequate nutrition during infancy and early childhood is a fundamental prerequisite of each child's full human developmental potential. Thus, the period from conception through birth to 2 years of age is a vital window for child optimal growth, health, and behavioural development.^2^ Literature shows that, undernourishment during the first 1000 days, from pregnancy through the child's second birthday, results to long term and irreversible growth damage, with impacts observed at the individual, community, and nation as whole.^3^ In the joint United Nations International Children's Emergency Fund (UNICEF), World Health Organization (WHO) and World Bank 2013 report, 162 million children under the age of five are affected by stunting globally either during pregnancy or after delivery; this being only a two-percentage-point lower than it was 5 years ago.^4,5^ If the current trend is to continue, projections in East Africa show that 42.1% of children under five years of age will be stunted in 2025.^6^ Studies have indicated that stunting can be inherited from one generation to the next.^7,8^ This may occur when an undernourished woman bears low birth weight babies, who if are subjected to suboptimal feeding practices and high rate of infectious diseases, do not experience the catch-up growth in subsequent years leading to an intergenerational cycle of stunting.^7^ United Nations International Children's Emergency Fund (UNICEF) describes four forms in which under nutrition can manifest: underweight (<-1SD weight-for-age Z-score), wasting (<1SDweight-for-height), and stunting (<-1SD height-for-age). In 2010 UNICEF reported among these 3 forms of under nutrition, stunting was globally higher - for the year 2015 the global prevalence of stunting was 42% in comparison to wasting and underweight which were 5% and 16%, respectively.^2^ A similar trend was observed in the Joint UNICEF, WHO and World Bank report of 2012 whereby 26% of the global under-fives population were stunted, 8% wasted and 11% underweight.^8^ The stunting was reported to be caused by the chronic lack of adequate nutritious food, poor child care practices, lack of access to health, and other social services.^8^

Forty-two percent (42%) of children are affected by stunting either during pregnancy or after delivery globally. The prevalence of stunting in Tanzania is estimated to be 35%.^3^ Kigoma region is one of the regions with a “very high” prevalence of stunting in children less than five years, estimated at 35 %.^2^

Bad feeding habits, number of household members, frequent infectious diseases attack, unhealthy environment, inadequate care practices, poverty, illiteracy, socio norms and Water, Sanitation, and Hygiene practices are determinants of stunting commonly described in most literature on stunting among under five years of age. We assumed that the distribution of these determinants may be different among school children as they were not under total parental care and can secure food on their own if available. They also may access food at school as well as other health interventions such as de-worming and schistosomiasis treatment. To ascertain whether these determinants affect primary school children in the same way as they do among under-five children, we conducted this study aiming at estimating the prevalence of stunting and its associated factors among primary school children in Kasulu District.

## MATERIALS AND METHODS

### Study Area and Design.

This was a cross-sectional study conducted in Kasulu District involving primary school pupils attending public schools by January 2018. Kasulu District Council was purposefully selected among the eight councils in Kigoma region, due to its high population density. Food adequacy in the district is determined by cross broader trade across the national border and/or inter-regional boundaries which have tended to shift large quantities of food across the border to neighbouring regions or countries. The most cultivated crops are maize, cassava, potatoes, and beans.^31^ Kasulu district has been the recipient of the highest number of refugees and asylum seekers from the Republic of Burundi and the Democratic Republic of Congo. The total district population was 556,851; 273,904 (49.2%) being male, 50.7% were children aged between 5-12 years as projected by the National Bureau of Statistics in the 2018 dashboard. The population growth was estimated at 2.4, primary school enrolment was about 59%, and an illiteracy rate of 23%.^32^ Kasulu district is highly populated in the region contributing to 20% of the region population with an average household size of 7.6.^9^ It also hosts more refugees than any other district in the region. The district has a total of 79 public primary schools with no private primary schools, 38 dispensaries, and 5 health centres.

### Sample Size Calculation

The sample size was calculated using Leslie's formula. The estimation of the sample size based on the stunting prevalence of 38% taken from a study done by *Semali et al*^1^^4^ in Kongwa District Tanzania, a site with similar settings as ours, with a marginal error of 5% and 95% confidence level. A response rate of 90% was used to adjust the estimated sample size resulting in a minimum sample size of 400 study participants.

The sample size (n) was calculated using Leslie's formula

$$\begin{array}{c} \text{n}={\text{Z}}^{2}\times \text{ P}\;\left(\underline{100-\text{P}}\right)\;\\ {\text{ε}}^{2}\\ \text{Where}\;\left(\text{n}\right)\;=\;\text{Sample}\;\text{size}\\ Z=1.96\;\left(95\%\;\text{confidence}\;\text{interval}\right)\;\text{in}\;\text{two}\;\text{tailed}\;\text{t}–\text{text}\\ \text{P}=\text{Prevalence}\;\text{of}\;\text{stunting}\;\text{of}\;38\%.^{14}\\ \text{ε}=\text{Marginal}\;\text{error}\;5\%\\ n=1.96^{2}\times \;38\%\;\text{x}\;\frac{\left(100-38\%\right)}{52}\;=362\;∼\;360\\ \text{Expected}\;\text{respone}\;\text{rate}\;\left(\text{RR}\right)\;=\;90\%\\ \text{Adjusting}\;\text{for}\;\text{non-}\text{response},\;\text{then,}\\ \text{n}=360/90\%\\ =400 \end{array}$$

Therefore, the minimum study sample size was 400 participants

### Sampling Technique

**FIGURE 1.**
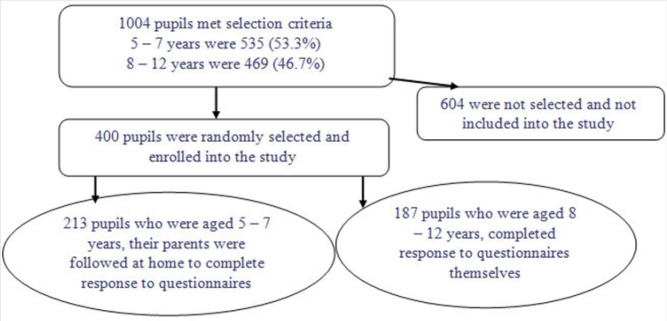
Study Participants’ Selection Framework

We used a systematic sampling technique to select three wards and one village from the respective ward. Kitema village, Kibirizi village, and Bugaga village were randomly selected from Nyenge Ward, Buhoro Ward, and Bugaga Ward respectively, and one public primary school from each village was randomly selected. Proportion probability sampling was used to estimate the sample size for each public primary school selected. The study respondents were stratified into two age groups: 5 to 7 years whose parents/guardians were traced at home to complete responses of the household questionnaire as their responses to some questions were not reliable; and 8 to 12 years who self-administered the questionnaire. (Figure 1)

The k^th^ interval depended on the number of eligible study participants in each school and each age strata. The formula below was used to calculate the kth interval

$$\begin{array}{c} \text{k}=\text{N}/\text{n} \end{array}$$

Where;

k is a sampling interval (sometimes called a skip)

N is the number of eligible study population size

n is the required sample size

### Data Collection Instruments and Procedures

A questionnaire was developed in English then translated to Swahili and back-translated to English to ensure a correct translation of meaning. Pre-testing of questionnaires was done to test for clarity of questions, validity, reliability, feasibility, and study logistics. This also assisted the research assistants to exercise flexibility in the wording of questions contained in a questionnaire. The pre-testing results were used to modify the content and wording of the questionnaire. The questionnaire had both closed and open-ended questions. The close-ended questions provided more structured responses to aid precision of responses to standardize analysis. The open-ended questions provided additional information that may not have been captured in the close-ended questions. The type of data collected included: socio-demographic and economic data, dietary practices, sanitation practices, school performance, and school attendance. The questionnaires were administered by a Principle researcher and two trained research assistants.

### Anthropometric Measurements

We measured height in centimetres to the nearest 0.1cm. The respondent stood upright on a uniformly calibrated height board facing forward without shoes. Height-forage Z scores were calculated for boys and girls separately. WHO 2006 references were used to define stunting – HAZ<-1SD was used to create the outcome variables.

### Data Management and Analysis

The responses to open-end questions were grouped then coded to respective codes in a questionnaire. The complete filled and coded questionnaires were entered in the Epi Info^TM^ make view; a trademark of the Centre for Diseases and Control, and then transferred to STATA software version 13 owned by StataCorp LP, 4905 Lake way Drive, College Station, Texas 77845, United States of America, for analysis. After data cleaning, frequencies, and percentages of independent variables were summarized in tables. Univariate, bivariate, and multivariable logistic regression models (inclusion criteria were any variable with p-value <0.2) were built to examine demographic characteristics and their association with stunting. We created population wealth quintiles by Principal Component Analysis. We reported Odd Ratios and their 95% Confidence Interval, and the significance level of alpha <5%.

### Ethical Considerations

We obtained ethical clearance to conduct the study from the Muhimbili University of Health and Allied Sciences Institutional Review Board. We sought permission to conduct this study from the Kasulu district authority. The head teacher signed written informed consent and parent/guardian consented on behalf of children. We explained study objectives, methodology, and benefits to the head teachers of respective primary school and parent/guardian of each study participant. Study participants aged 8 years and older assented before recruitment into the study. All participants were free to withdraw from the study at any point in time. We assigned numbers to participants to assure confidentiality and only the researcher and research team accessed the respondent questionnaires

## RESULTS

### Demographic Characteristics of the Study Population

We enrolled a total of 400 pupils with a mean age of 7.51 (SD = 1.54) years with a 100% respondents response rate to the study. More than half 213(53.2%) were aged between 5 to 7 years, and about half 201(50.3%) were boys. More than half of the respondents 201 (50.3%) were in class two, while 123 (30.8%) were in class one and 76 (19.0%) were in class three. About forty percent of 159 (39.8%) of the pupils were from Kibirizi primary school. The mean age of the parents/guardians was 32.4 (SD =5.09) years, with a majority of 243 (60.8%) aged between 26–35 years. More than half of the parents/guardians 211 (52.3%) had primary education, while only 50 (12.4%) had secondary education or above. The majority of 248 (62%) of the parents/guardians were either peasants or livestock keepers or about 1 in 5 were involved in petty business 86 (21.5%), 57 (14.3%) were skilled labourers and only 9 (2.2%) were employed.

Almost all parents/guardians were married or cohabiting 366 (91.5%) (Table 1)

**TABLE 1:** Demographic Characteristics of Pupils and their Parents/Guardians

Pupils characteristics			Parent/guardian characteristics		
Variable	N	%	Variable	N	%
**Age, Years**			**Age, Years**		
Mean (SD1)	7.5	1.54	Mean (SD)	32.4	5.09
5 – 7	213	53.3	<26	31	7.7
8 – 12	187	46.8	26 – 35	243	60.8
**Sex**			>35	126	31.5
Girls	199	49.8	**Marital status**		
Boys	201	50.3	Not married^*^	34	8.5
**Class**			Married/cohabiting	366	91.5
One	123	30.8	**Education**		
Two	201	50.3	No formal education	139	34.8
Three	76	19	Primary education	211	52.8
**School**			Secondary and above	50	12.4
Kitema	125	31.2	**Occupation**		
Kibirizi	159	39.8	Peasant/livestock keepers	248	62
Bugaga	116	29.0	Petty business	86	21.5
			Skilled labourers	57	14.3
			Employed	9	2.2

^*^ Mean (Standard Deviation), ^**^ Single variable comprised 'divorced, separated and single

### Prevalence of Stunting Among Study Respondents

**FIGURE 2.**
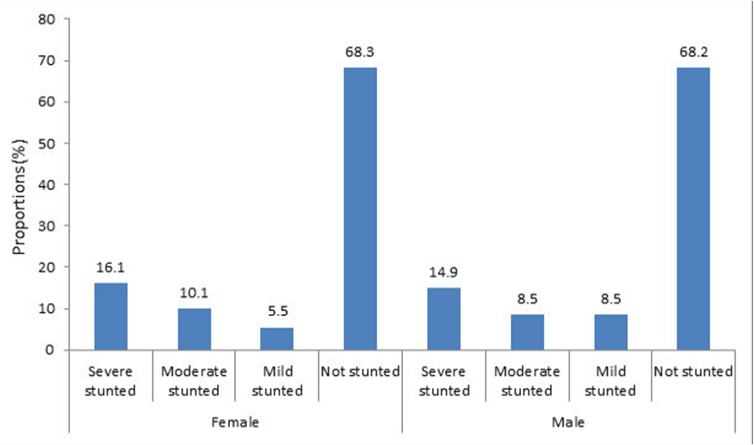
Prevalence of Stunting by Sex in Public Primary School Pupils in Kasulu District

The overall prevalence of stunting was 127 (31.8 %) (95% CI: 27.2%–36.6%), where 63 (31.7%) girls and 64 (31.8%) boys were considered as stunted. The distribution of stunting among girl's pupils was 5.5% mild, 10.1% moderate, and 16.1% severely stunted; and boys were 8.5% mild, 8.5% moderate, and 14.9% stunted. (Figure 2).

### Socio-Economic Determinants of Stunting

Older pupils 8-12years of age were significantly more likely to be stunted 70 (37.4%) as compared to younger pupils 5-7 years 57 (26.8%; p=0.022). Stunting was not differentially distributed by sex (boys 64 (31.8%) and girls 63 (31.7%; p = 0.969). Pupils in lower classes one 41 (33.3%) and two70 (34.8) were more stunted than pupils in class three 16 (21.1%; p = 0.081).

Older parents/guardians (>35 years) had a higher probability of 76 (60.3%) of having a child who was stunted than parents/guardians aged 26-35 years 44 (18.1%) or parents/guardians age<26 years 7 (22.6%; p<0.001). Having no formal education had a higher likelihood of 62 (44.6%) of having a stunted child than primary education 56 (26.5%) and secondary education or higher 9 (18.0%; p<0.001). Being either a peasant or livestock keeper was associated with a significantly higher probability of having a stunted child 91 (37.5%) compared to those who were employed 2 (22.2%), skilled labourers 9 (15.8%) or in petty business 25(28.4%; p = 0.014).

Parents/guardians who were categorised as not being in marriage (single, separated, divorced, or widow) had a significantly higher likelihood of having a stunted child than those who were married or cohabiting 24 (70.6%) vs. 103 (28.1%; p<0.001). A household, which had six or more children, were significantly more likely to have a stunted child 73 (59.8%) than households with five or less 54 (19.4%; p<0.001). Also, pupils not living with both of their parents (categorized as others) had a higher proportion of stunting compared to those who reported living with both parents 31 (68.9% vs. 96 (27.0%; p<0.001). Households ranked as having lower economic status were significantly associated with stunting than households ranked in the highest economic status where 3 (3.8%), 11 (14.1%), 18 (22.0%), 42 (53.2%), 53 (65.4%) percent of the children were categorised as stunted from lowest, low, middle, high and highest quintile, respectively (p trend < 0.001)

### The Influence of Dietary Practices on Stunting

Pupils reporting coming from households with inadequate food were more likely to be stunted compared to pupils coming from households reported to have adequate food supply 97 (84.4%) vs. 30 (10.5%; p<0.001). Reporting eating less than 2 meals a day had a 108 (61.7%) probability of stunting compared to 19(8.4%) in pupils reporting eating 3 meals (p < 0.001). Stunting was not differentially distributed by food diversity (starch only 65 (32.3%), starch and protein 23 (26.1%), starch protein and fats 6 (33.3%) and starch, protein, fats and vegetables/fruits 33 (35.5%; *p=.587*). Pupils reported eating proteins’ food less frequently had a higher chance of being stunted than children who reported eating proteins’ food frequently (*p<.001*). There was no difference observed between sharing 103 (31.4%) and not sharing plate 24(33.3%) during eating (*p=.750*).

### The Influence of Hand Hygiene Practices to Stunting

Not washing hands after visiting the toilet at school increased the chance of stunting by 103 (52.3%) in comparison with washing hands with running water only or with soap which was 24(11.8%; p<0.001). Similarly, not washing hands at home after visiting the toilet was associated with a 39(92.9%) increased probability of stunting compared to washing hands with running water only or with soap 88(24.6%; *p<.001*)

### Multivariable Analysis of Stunting By Social-Economic, Dietary, Hygiene, and Sanitation Factors

Socio-demographic characteristics that were significantly associated with stunting in this population were household wealth quintiles, household food security, and frequency of eating proteinous food and hand wash practices at both school and home. Household wealth had a linear relationship with stunting where pupils from the lowest quintile had a 28 fold increase chance of being stunted (AOR = 28; 95% CI: 3.64 – 214.6; p<0.001) followed by the 2nd quintile (AOR = 17; 95%CI: 2.20–138.5; p<0.01), the 3rd quintile(AOR = 8.0; 95%CI: 0.99 –64.67; *p=.051*) and 4th quintile (AOR=4.2; 95%CI: 0.49–6.75; *p=.191*) when compared to 5th (highest) wealth quintile.

Pupils who reported inadequate food in the household had 11 times more likelihood of being stunted than pupils who reported adequate food in their households (AOR = 10.6; 95%CI: 4.60 – 24.60; p< 0.001). The probability of being stunted increased by a factor of 14 times if the eating of proteinous food occurred once a month or less compared to those who ate proteinous foods 3 times per week (AOR = 14.6; 95%CI: 4.07 – 52.42; *p<.001*).

Improper hands wash after toilet both at school and the home was significantly associated with stunting. Those who reported not to wash hands after toilets at school were at higher risk of being stunted (AOR = 3.5; 95%CI: 1.62 – 7.58; *p=.001*), compared to those who used either water only or with soap for washing hands after toilets. At home pupils who reported not to wash hands were at 13 folds increase at risk of being stunted (AOR =13.0; 95%CI: 2.73 – 61.76; *p=0.001*)

### Relationship between Stunting and School Attendance and Academic Performance

Pupils who were stunted had a higher proportion of irregular school attendance 35 (27.6%) compared to pupils who were not stunted 10(3.7%; *p<.000)*. Being stunted 68(77.3%) was associated with a significantly higher chance of failing at the end of year examination compared to those who were not stunted 13(6.7%; *p<0.001*).

### Multivariable Analysis of School Attendance and Academic Performance by Stunting

Stunted pupils were likely to have irregular school attendance (AOR = 9.4; 95%CI: 4.42 – 19.93; *p<.001)-*examination (AOR = 3.9; 95%CI: 1.67 – 8.92; *p<.01)*.and failed in recent end of year examinations (AOR=23.6; 95%CI:10.24–54.19; *p<.001*). Older age pupils were likely to have irregular school attendance (AOR=3.7; 95%CI:1.73–7.73; *p<.001*) as compared to younger pupils. And pupils who belonged in a household with inadequate food were likely to fail at the end of year

**TABLE 2:** Logistic Regression Analysis of Stunting by Social Economic, Dietary, Hygiene and Sanitation Factors

Variables	Crude OR (95%CI^a^)	Adjusted OR (95%CI^b^)
**Age, in years**		
5 – 7	Ref	
8 – 12	^**^1.6 (1.07 – 2.50)	NA
**Sex**		
Girls	Ref	
Boys	1.0 (0.66 – 1.54)	NA
**Class of the pupils**		
One	^*^1.9 (0.96 – 3.65)	NA
Two	^**^2 (1.07 – 3.74)	NA
Three	Ref	
**Parent/guardian characteristics**		
**Age, years**		
<26	1.3 (0.53 – 3.25)	NA
26 – 35	Ref	
>35	6.9 (4.24 – 11.15)^***^	NS
**Parent/guardian education**		
No formal education	3.7 (1.66 – 8.12)^***^	NS
Primary education	1.6 (0.75 – 3.60)	NS
Secondary & high education	Ref	
**Parent/guardian Occupation**		
Peasants/Livestock keepers	3.1 (1.47 – 6.68)^**^	NS
Petty business	2.1 (0.91–4.95)	NS
Skilled labour	Ref	
Employment	1.5 (0.27 – 8.55)	NS
**Marital status**		
Not married	6.1 (2.83 – 13.26)^***^	NS
Married/cohabiting	Ref	
**Number of children in household**		
5 or less	Ref	
6 or more	^***^6.2 (3.87 – 9.87)	NS
**A person living with a child**		
Both parents	Ref	
Others	6.0 (3.05 – 11.711)^***^	NS
**Household wealth quintiles**		
Lowest	48.6 (14.05 – 168.05)^***^	28.0 (3.64 – 214.6)^***^
Low	29.1 (8.47 – 100.10)^***^	17.4 (2.20 – 138.5)^**^
Medium	7.2 (2.03 – 25.61)^**^	8.0 (0.99 – 64.67)
High	4.2 (1.12 – 15.74)^**^	4.2 (0.49 – 36.75)
Highest	Ref	Ref
**Household food security**		
Adequate	Ref	Ref
Inadequate	45.8 (24.41 – 85.95)^***^	10.6 (4.60 – 24.60)^***^
**Number of meals per 24 hours**		
Two or less	17.5 (9.98 – 30.60)^***^	NS
Three	Ref	
**Frequency of eating protenous food**		
Three times per week	Ref	Ref
Once per week	1.4 (0.63 – 3.14)	1.1 (0.37 – 3.30)
More than once per month	21.3 (10.90 – 41.74)^***^	3.0 (1.14 – .17)^**^
Once or less than once per month	59.2 (22.60 – 155.0)^***^	14.6 (4.7 – 54.42)^***^
**Hand wash practices after visiting toilet at school**		
Do not wash hands	8.2 (4.91 – 13.60)^***^	3.5 (1.62 – 7.58)^***^
Wash with water only or with soap	Ref	Ref
**Hand wash practices after visiting toilet at home**		
Do not wash hands	39.9 (12.03–132.0)^***^	13.0 (2.73–6176)^***^
Wash with water only or with soap	Ref	Ref

a Odds Ratio (95% Confdence Interval);

b Adjusted Odds Ratio (95% Confdence Interval) for all variables in the table;

^*^ p value <0.05; ^**^ p value < 0.01; ^***^ p value < 0.001; NA = Not applicable; NS = Not signifcant

**TABLE 3:** Logistic Regression Analysis Academic Performance and School Attendance by Stunting Status and Socio-Demographic Factors

	School attendance Crude OR (95% CI)	Adjusted OR (95%CI)	Academic performance Crude OR (95%CI)	Adjusted OR (95%CI)
**Stunting status**				
Not stunted	Ref	Ref	Ref	Ref
Stunted	10	9.4	47.3	23.6
	(4.77 – 21.0)^***^	(4.42 – 19.93)^***^	(22.3 – 100)^***^	(10.24-54.19)^***^
**Household food security**				
Adequate	Ref		Ref	Ref
Inadequate	6.3	NS	17.8	3.9
	(3.26 – 12.37)^***^		(9.37 – 33.7)^***^	(1.67-8.92)**
**Pupils age group in years**				
5 – 7	Ref			
8 – 12	4.1	3.7		
	(2.0 – 8.31)^***^	(1.73 – 7.73)^***^	NA	
**Number of meals per 24 hours**	5.4		8.5	
Two or less				
	(2.58 – 11.20)^***^	NS	(4.56 – 15.83)^***^	NS
Three	Ref		Ref	
**Pupil's class**				
One	Ref 4.5			
Two	(1.69 – 11.8)** 2.8	NS	NA	NA
Three	(0.87 – 8.83)	NS	NA	NA
**Frequency of eating protenous food**				
Three times per week	Ref		Ref	
Once a week	1.3		1.3	
	(0.48 – 3.52)	NS	(0.53 – 3.23)	NS
> Once per month	3.0		9.1	
	(1.32–6.62)**	NS	(4.41–18.84)^***^	NS
Once or < once per month	^***^4.6		24.2	
	(1.98 – 10.8)	NS	(9.12 – 64.19)^***^	NS
**A person living with a child**				
Both parents			Ref 2.8	
Others	NA	NA	(1.35 – 6.02)^*^	NS
**Household wealth quintiles**				
Lowest	4.0		16.3	
	(1.25 – 12.65)^*^	NS	(4.61 – 57.84)^***^	NS
Low	4.5		14.4	
	(1.41 – 14.09)^*^	NS	(4.04 – 51.0)^***^	NS
Medium	2.6		4.8	
	(0.79 – 8.79)	NS	(1.29 – 18.06)^***^	NS
High	0.5		1.5	
	(0.89 – 2.81)	NS	(0.32 – 7.17)	NS
Highest	Ref		Ref	

a Odds Ratio (95% Confdence Interval);

b Adjusted Odds Ratio (95% Confdence Interval) for all variables in the table;

^*^ p value < 0.05; ^**^ p value < 0.01; ^***^ p value <0.001, NS = Not signifcant, NA = Not applicable

## DISCUSSION

This study aimed to determine the prevalence of stunting and its associated factors among public primary school pupils in Kasulu District. We observed that 31.8% of the study population was stunted, in which 49.6% were girls and 50.4% were boys, showing both girls and boys being equally affected by stunting. Also, inadequate household food supply; reduced frequency of eating proteinous food, and poor hand hygiene after toilet use significantly were associated with stunting. Importantly, stunting negatively affected school attendance and subsequent pupil's academics. It has shown that inadequate food at home reduce children learning capacity at school.

### Prevalence of Stunting

This prevalence of stunting is high and according to WHO is of public health concern requiring interventions. We observed a 6% higher prevalence than the global prevalence of stunting among under-five children which is at 26%, ^8^ but, was about 6% lower than the African region stunting prevalence^9^, and lower than that found by *Galgamuwa et al.*^16^

Other studies done in the African region among a similar population have reported varying prevalence of stunting. For instance, *Biadgilign et al.*^17^ and *Derso T, et al* ^11^ found the prevalence of stunting to be 46.7% and 58.1%, respectively while *Bamba I, et al* reported a prevalence of 29.4% among primary school children.^12^ Differences in prevalence were attributed to differences and similarities in study methodology, study age population, and the difference in study geographical areas.

Besides, this prevalence is 3% lower than the estimated stunting prevalence among under-fives in Tanzania and 2% lower than what was found in THDS-MIS 2015-2016.^33^ suggesting that stunting affects primary school pupils at the same magnitude as it affects children under five years of age.

Similarly, *Masanyiwa et al* in Nzega Tanzania found a prevalence of 26.1%, and *Semali et al* found a prevalence of 47.9%,^13^, ^14^ these studies were done among under-fives.

We observed that stunting affected public primary pupils as it was observed among under-five children. We found insignificant variation of stunting between Kasulu District public primary school and other parts of Tanzania. This prevalence is high and poses challenges to Kasulu District public health to achieving Sustainable Development Goal. It also has a negative implication for the nation, as stunting was associated with poor academic trajectory.

### Socio-Economic Factors

In our study household wealth was found to be a significant factor associated with stunting. As the household wealth quintile increases, the risk for stunting decreases. This corroborates with findings in a study done by *Makoka et al, Semali et al and Masanyiwa et al.*^18^, ^14^, ^13^.

It also concurs with findings in the studies done in African countries and other parts; *Herrador et al.*^3^^4^, and the study done in Sri-Lanka by *Galgamuwa et al.*^16^

### Dietary Practices

We found that inadequate household food security was associated with stunting. A similar observation was observed by *Nyaruhucha et al,*^22^ in Simanjiro district Tanzania reported that 87% of stunted children came from households with food insecurity of which food was described as poor in terms of both quantity and quality.^22^ However, food diversity and plate sharing during eating on multivariable analysis were not associated with stunting as it was found in a study done by *Masanyiwa et al,*^13^ *Semali et al,*^14^ and *Chirande et al,*^19^ the difference can be explained by the difference in the age group of study populations and study methodology

### Water, Sanitation, and Hygiene

Additionally, we found that; stunting among primary pupils was associated with poor hand hygiene after visiting toilets. This habit may have predisposed them to water-borne diseases and to intestinal worm infestation and diarrhoea diseases, which if left untreated deprives nutrients and chronic deprivation lead to stunting. Similar results were found by *Masanyiwa et al,*^13^ in Nzega, Tanzania, *Biadgilign et al*,^17^ in Ethiopia, *Kofuor et al*,^25^ in Ghana.

### Academic Performance/Attendance

We observed that majority of stunted children had high odds of performing poorly in the recent overall end of year examinations. Similar observations were found among school children in Malaysia,^21^ in North Tripura,^22^ Ethiopia,^18^ and Burkina Faso.^23^ The impact of stunting on academic performance has negative onward academic trajectories and well-being. We further observed that; older and stunted pupils had irregular school attendance; however, pupil's age was not associated with stunting. These findings were like findings found by SR NAIK et al and Stuber et al.^24^, ^25^

### Study Limitations

Some study participants did not understand Kiswahili which necessitated finding interpretation which may have resulted in misclassification biases. Data collection was done in the rainy season where vegetables and fruits were readily available which may have shifted the study results to either direction, and about 30% of study respondents were in class one who had not experienced school life hence not exposed to starvation for a long time during class hours which may have affected stunting prevalence.

Academic performance was examined for about 70% of respondents as class one pupils had no examination results, this may have affected the association

## CONCLUSION

The study was able to determine the socio-economic factors which were associated with stunting. It was found that household wealth predetermined a pupil's stunting status. Moreover, household food insecurity and not including protein-containing food in daily meals contributed significantly to pupils stunting. Also, poor hand wash after visiting the toilet was found to be associated with stunting. More stunting has a significant association with school academic performance and attendance.

### Recommendations

Primary schools based programs to address stunting such as school feeding program should be implemented and strengthened, making use of locally available foods, coupled with creating community awareness on the inclusion of food diversity in a meal, strengthening water, sanitation, and hygiene in schools, and channel the community program such as Tanzania Social Action Funds (TASAF) to the household with food insecurity by emphasizing on micro-agro-business to supplement household with food insecurity.

Other researches with superior methods should be done in Kasulu District to establish causality, and to find out whether the stunted pupils received or missed nutrition interventions during under-fives and how to mitigate factors associated with stunting among public primary school pupils.
